# Attachment Representations in Children with and without Attention-Deficit/Hyperactivity Disorder (ADHD)

**DOI:** 10.3390/brainsci11111516

**Published:** 2021-11-16

**Authors:** Tycho J. Dekkers, Rianne Hornstra, Barbara J. van den Hoofdakker, Suzanne R. C. de Jong, Jessica V. Schaaf, Guy Bosmans, Saskia van der Oord

**Affiliations:** 1 Department of Child and Adolescent Psychiatry, University Medical Center Groningen, University of Groningen, 9713 GZ Groningen, The Netherlands; r.hornstra@accare.nl (R.H.); b.van.den.hoofdakker@accare.nl (B.J.v.d.H.); 2 Accare Child Study Center, 9713 GZ Groningen, The Netherlands; 3 Academic Center for Child and Adolescent Psychiatry, Levvel, 1105 AZ Amsterdam, The Netherlands; 4 Department of Psychology, University of Amsterdam, 1018 WS Amsterdam, The Netherlands; j.v.schaaf@uva.nl; 5 Department of Child and Adolescent Psychiatry, Amsterdam University Medical Centers (AUMC), 1105 AZ Amsterdam, The Netherlands; 6 Department of Clinical Psychology and Experimental Psychopathology, University of Groningen, 9712 TS Groningen, The Netherlands; 7 Clinical Neuropsychology Section, Department of Clinical, Neuro and Developmental Psychology, Vrije Universiteit, 1081 HV Amsterdam, The Netherlands; s.r.c.dejong@vu.nl; 8 Faculty of Psychology and Educational Sciences, KU Leuven, 3000 Leuven, Belgium; guy.bosmans@kuleuven.be (G.B.); saskia.vanderoord@kuleuven.be (S.v.d.O.); 9 Leuven Brain Institute, 3000 Leuven, Belgium

**Keywords:** Attention-Deficit/Hyperactivity Disorder (ADHD), attachment, children, parenting, story stem, parent–child relationship

## Abstract

Attention-Deficit/Hyperactivity Disorder (ADHD) in children is associated with several adverse family characteristics, such as higher parenting stress, more conflicted parent–child relationships, lower parental competence, and higher levels of parental psychopathology. Hence, children with ADHD more often grow up under suboptimal circumstances, which may impact the development of their attachment representations. Here, we investigated whether children with ADHD have more insecure and disorganized attachment representations than their typically developing peers, and which factors could explain this association. We included 104 children between 4 and 11 years old, 74 with ADHD (without Conduct Disorder) and 30 typically developing control children. Children completed a state-of-the-art story stem task to assess their attachment representation, and we measured parents’ expressed emotion (as an index of parent–child relationship quality), parents’ perceived sense of competence, parental education levels, and parent-rated ODD symptoms of the child. We found that, after controlling for multiple comparisons, children with ADHD had less secure and more ambivalent and disorganized attachment representations relative to their typically developing peers. These group differences were independent of comorbid ODD and parental education levels. There were no group differences on avoidant attachment representations. Explorative analyses within the ADHD group showed that attachment representations were not related to parent–child relationship quality, perceived parenting competence, parental education levels, and comorbid ODD symptoms. We conclude that children with ADHD disproportionately often have attachment problems. Although this conclusion is important, treatment implications of this co-occurrence are yet unclear as research on ADHD and attachment is still in its infancy.

## 1. Introduction

Attention-Deficit/Hyperactivity Disorder (ADHD) is a neurodevelopmental disorder characterized by age-inappropriate levels of inattention, hyperactivity, and/or impulsivity, causing impairment in several domains of life [1]. In children, the prevalence of ADHD is high, with 5–7% of children meeting diagnostic criteria [2,3]. In families of children with ADHD, parenting stress is higher, parenting competence is lower, there is more parental psychopathology, parent–child relationships are more often conflicted, and the socioeconomic situation is disadvantageous relative to families of children without ADHD [4,5,6]. This suggests that children with ADHD disproportionately often grow up under adverse family conditions. As adverse family conditions often hinder the development of secure attachment [7], the link between ADHD and adverse family conditions has guided the hypothesis that children with ADHD more often have insecure and/or disorganized attachment representations. The current study therefore sets out to investigate whether school-age children with ADHD are less inclined to develop secure or less organized attachments than their typically developing peers.

Generally, three different attachment representations are distinguished. Sensitive and supportive parenting in response to stress will help the child to develop a secure base script, ultimately leading to a secure attachment representation [8]. On the other hand, insecure attachment develops when children have a lack of trust in the caregivers’ availability when in need of protection or support. This can cause ambivalent or avoidant attachment representations, in which children either focus their attention exclusively on their caregivers or avoid their caregivers and rely on themselves. Finally, some children do not show coherence in their attachment behaviors (i.e., demonstrating disoriented or contradictory behaviors), which is classified as disorganized attachment [9]. Insecure and disorganized attachment has been linked to multiple forms of psychopathology, ranging from internalizing to externalizing disorders (see [10,11,12] for meta-analyses).

Despite its potential relevance, empirical research on the link between attachment and ADHD is scarce. A recent meta-analysis including 62 studies found a link between children’s attention problems and attachment quality: children with more insecure and children with more disorganized attachment representations had more attention problems than children with secure or organized attachment representations, although the magnitude of the effects was modest [13]. A systematic review similarly concluded there is an association between ADHD and insecure attachment [14]. Upon further inspection of the studies included in these reviews, three significant limitations of the literature on ADHD and attachment emerged.

First, most studies investigated the link between attachment and ADHD symptoms in samples of typically developing children, or in children at risk for attachment problems [13]. Only a small proportion of the studies reviewed utilized a case-control design by comparing children with and without clinical diagnoses of ADHD, which is particularly necessary to estimate the relevance of attachment for children with ADHD. Generally, these case-control studies confirmed that children with ADHD had more insecure attachment patterns than their peers without ADHD [15,16,17,18,19], although some studies did not find such an association [20,21].

Second, many studies investigating the link between ADHD and attachment did not control for comorbid disruptive behavior problems. Abundant literature shows a significant association between disruptive behavior and insecure attachment [2,10]. The relevance of controlling for disruptive behavior is further demonstrated by a study that found large differences in attachment representations between children with and without ADHD, which disappeared after controlling for disruptive behavior problems [20]. As ADHD and disruptive behavioral disorders often co-occur [22], and disruptive behavior disorders and insecure attachment are associated [2,10], controlling for disruptive behavior is imperative when assessing the link between ADHD and attachment [23].

A third limitation of the literature on ADHD and attachment relates to the measurement of attachment. From school-age, observational methods such as the Strange Situation Test [8] are less applicable because attachment *behavior* has become too subtle [24]. In addition, the scoring of insecure/disorganized attachment behavior may be confounded with ADHD characteristics using these measures, as behavioral (hyper)activity and rapid shifts in attention could be related to both [21,25]. Many studies on school-age children used self-report measures, which have different disadvantages: (i) self-report measures could invoke social desirability, especially on sensitive topics such as parent–child relationships; (ii) self-report is particularly unreliable in children with ADHD and/or Oppositional Defiant Disorder (ODD) [26]; (iii) a positive illusory bias is often reported in children with ADHD, meaning that children with ADHD systematically overestimate their own capacities [27]. A solid and well-established alternative to observational and self-report measures of attachment are representational measures of attachment, such as story stem procedures, in which children’s cognitive attachment *representations* are derived from the completion of a story by the child [24,28].

In the current study, we take these limitations of previous studies into account, by investigating attachment representations in a carefully diagnosed sample of school-age children with and without ADHD, matched by age, sex, and intelligence, controlling for comorbid disruptive behavioral symptoms and using state-of-the-art story stem procedures to measure attachment representations. We hypothesize that children with ADHD have more insecure and more disorganized attachment representations, relative to typically developing children, and—based on the available literature on ADHD and insecure attachment—that this cannot be entirely explained by comorbid disruptive behavior. Then, we zoom into the group of children with ADHD to explore whether insecure and/or disorganized attachment representations in these children could be accounted for by parents’ expressed emotion (as indicator of parent–child relationship quality), parents’ perceived sense of competence, parental education levels, and children’s comorbid ODD symptoms. Ultimately, this may guide interventions for children with ADHD and their families.

## 2. Materials and Methods

### 2.1. Participants

A total of 74 children with ADHD (combined presentation: *N* = 44, inattentive presentation: *N* = 21, hyperactive/impulsive presentation: *N* = 9) and 30 Typically Developing (TD) children participated. Children with ADHD were recruited for a microtrial on behavioral parent training in six child mental healthcare outpatient clinics in the Netherlands [29]; TD children were recruited from the community. All children were between 4 and 11 years old and had an IQ of at least 70. Children included in the ADHD group were all previously diagnosed with ADHD in routine clinical care (which involves multi-informant assessment following evidence-base guidelines) and in addition had to meet criteria for any ADHD presentation on the Diagnostic Interview Schedule for Children (DISC-IV) [26]. Children were included in the TD group when not meeting criteria for ADHD on the same measure. None of the children were taking stimulant medication; children with an autism spectrum disorder or conduct disorder (CD) were excluded. Medical ethical approval was waived by the Medical Ethical Committee of the University Medical Center Groningen (METc2016/197).

### 2.2. Measures

#### 2.2.1. ADHD/ODD/CD

The Disruptive Behavior Disorders module of the Dutch version of the structured Diagnostic Interview Schedule for Children (DISC-IV) [30] was administered to one of the parents/caretakers. This interview follows DSM-IV symptoms of ADHD, oppositional defiant disorder (ODD), and CD. Test–retest reliability and predictive validity (i.e., agreement of DISC-IV diagnoses with clinicians’ ratings) of the DISC-IV are good (*κ* = 0.79 and *κ* = 0.72, respectively) [26]. The DISC was used as a diagnostic measure (i.e., to include children with ADHD and to exclude children with CD), as well as to measure the number of ODD symptoms.

#### 2.2.2. Intelligence

Two subtests (Block Design and Vocabulary) of the Dutch Wechsler Intelligence Scale for Children (WISC-III) [31,32] or, with children younger than 6 years old, the Dutch Wechsler Preschool and Primary Scale of Intelligence (WPPSI-III) [33,34], were administered to estimate intelligence. These subtests correlate highly with full-scale intelligence quotients [35,36]. In case there was a recent intelligence score in the child’s patient file, we used that score in order to not bother children unnecessarily.

#### 2.2.3. Parental Education Level

Parent’s highest level of education (8-point scale, with higher values indicating higher levels of education) was obtained. We could not obtain this information for two participants.

#### 2.2.4. Attachment Representations

We used a story stem task to assess attachment representation. In this task, the researcher starts telling four stories to the child while using two dolls (one representing the child and the other the parent, gender and ethnicity matched) and a standardized set of attributes. Each story has an attachment-related theme, and the child is asked to finish all stories [24]. The themes of the four stories were “separation from parent” and “reunion with parent” (for all children), “hurt knee” and “monster in the bedroom” (for children 4–8 years old), and “in need of school assistance”, and “fight with friend” (for children 8–12 years old). The task was originally developed as the Doll Story Completion Task to establish attachment in three-year-old children [37], and was adapted to assess attachment in middle childhood [28]. We used the task by Kerns and colleagues [24,38], who made three noticeable changes relative to the task developed by Granot and Mayseless: (i) some of the original stories did not elicit attachment themes when used in the United States, and were therefore replaced by new stories; (ii) stories were made less hypothetical by replacing fictive names by names of the child and his/her actual family members, and (iii) scoring was eased by removing siblings from the stories. Psychometric properties of the story stem task as established in previous studies were good: stability was moderate to high, and convergent validity was good as outcomes were related to questionnaire-based attachment measures, and to maternal behavior, child adjustment, peer relationships, emotion regulation, and academic adjustment [38]. Note that each story stem dimension is strongly linked with the same label category in Ainsworth’s [8] coding system in such a way that children scoring high on the dimension are most similar to the description of the category.

The session was recorded, and all tapes were scored independently afterwards by two raters (R.H. and S.d.J., interrater reliability *κ* = 0.82 − 0.96). Raters followed a training in the assessments and scoring of the tapes. All disagreements were solved after discussion. Ultimately, this resulted in a score between 1 (no signs) and 5 (prototypical) on each of the four attachment representations (secure, ambivalent, avoidant, disorganized).

#### 2.2.5. Parent–Child Relationship: Expressed Emotion

Five Minute Speech Samples (FMSS) [39] were collected of parents of all children in the ADHD group as a measure of Expressed Emotion (EE). EE reflects the quality of the parent–child relationship, and reliably predicts negative outcomes [40]. Parents were asked to talk about their child for five minutes, without any interruptions of the researcher. When parents stopped talking before the five minutes were finished, the researcher encouraged the parents to continue. Based on previous literature [39,41], the FMSS yielded ratings on the initial statement (positive, neutral, or negative), warmth (high, moderate, or low), and relationship (positive, neutral, or negative), as well as the frequency of positive and critical comments. A total score of the ratings on the initial statement, warmth, and relationship was computed and served as the main dependent variable used in the analyses. If at least one of the ratings was low/negative, and the frequency of critical comments was higher than the positive comments, EE was considered high [39]. The speech sample was audio-taped and transcribed verbatim by research assistants. Two researchers (R.H. and S.d.J.) coded the first 20 transcripts independently from each other to establish reliability of the scoring; disagreement was discussed with senior author S.v.d.O., who completed specialized training in scoring of EE. Inter-rater reliability for the categorical ratings (initial statement; *κ* = 1.00, warmth; *κ* = 0.82, and relationship; *κ* = 1.00) and intraclass correlation coefficients for the frequency of positive/critical comments (ICC_negative_ = 0.974, ICC_positive_ = 0.945) were excellent.

#### 2.2.6. Parents’ Perceived Sense of Competence

The Parenting Sense of Competence scale (PSOC) [42] was used to assess parents’ perceived competence as a parent. The PSOC consists of 17 items (e.g., “being a parent is manageable, and any problems are easily solved”), which are answered on a 6-point Likert scale ranging from 1 (strongly disagree) to 6 (strongly agree). Higher scores indicate higher parenting sense of competence. Higher ratings of child problems were related with lower scores on the PSOC, and psychometric properties (factor structure, internal consistency) were adequate [43].

### 2.3. Procedure

Eligible parents were provided with an information letter. When interested, procedures were explained and parents provided informed consent. Parents received an online questionnaire with demographic questions and the PSOC. The remaining measures were administered during a home visit or a visit to the clinic, in which the parents and the child were assessed simultaneously for about two hours. Parents received compensation for participating (EUR 10).

### 2.4. Data-Analytic Approach

As preliminary analyses, we used independent t-tests, Mann–Whitney tests (in case of non-normal distribution of data), and chi-square analyses to assess between-group differences on age, sex, intelligence, parental education levels, and ADHD and ODD symptoms.

As primary analyses, to test whether children with and without ADHD differ in attachment representations, we planned to use an ANCOVA with group (ADHD vs. TD) as independent variable and the continuous score on the attachment representation as dependent variable. However, as Levene’s and Bartlett’s test both indicated unequal variances (in combination with our unequal sample size), we instead performed multilevel regression analyses with group as fixed effect in which we freely estimated group variances in attachment representations. As we had four measures of attachment representations, this analysis was performed four times, once for each attachment representation.

We included age as covariate as the literature shows attachment and age are correlated [44]. However, we did not include IQ, ODD symptoms, and parental education levels as covariates because literature reports these variables are not only related to attachment but also to ADHD [22,45,46]. As such, adding these variables to the regression model would result in removing meaningful variance from the effect of ADHD, making them unsuitable as covariates (for statistical background, see [47]).

Instead, to understand whether associations between ADHD and attachment were independent of ODD symptoms or parental education levels, we performed separate analyses with ODD symptoms and parental education level as independent variables instead of group (note that these were bootstrapped regression analyses instead of multilevel regression analyses as ODD symptoms and parental education levels were measured continuously and thus homogeneity of regression slopes is not assumed).

As secondary analyses, to elucidate which children with ADHD are particularly at risk for insecure and disorganized attachment representations, we used bootstrapped (as assumptions were violated) hierarchical linear regression analyses to assess the influence of age and parental education levels (block 1), comorbid ODD symptoms (added in block 2), expressed emotions, and parenting sense of competence (both added in block 3) *within* the group of children with ADHD. Participants were excluded from specific secondary analyses when data were missing for any of the relevant measures (note: we had missing data on the PSOC for 7 participants and on the FMSS for 6 participants).

In both sets of analyses, we applied a Benjamini–Hochberg correction [48] for multiple testing because we used four indices of attachment. We conducted all analyses in SPSS (v.25).

## 3. Results

### 3.1. Preliminary Analyses

Groups did not differ in age, sex distribution, and intelligence (Table 1). Parental education level was higher for the TD children than for the children with ADHD. As expected, children with ADHD had more ODD symptoms than TD children.

### 3.2. Primary Analyses: Group Differences

Multilevel regression analyses with age as covariate revealed that children with ADHD differed significantly from TD children on three of the four attachment representations. Children with ADHD had lower scores on secure attachment representations and higher scores on ambivalent and disorganized attachment representations relative to TD children. Groups did not differ in avoidant attachment representations. Statistics on group differences are depicted in Table 2. Age was not significantly associated with any of the attachment representations.

Importantly, we found no significant effects on any attachment representation when ODD symptoms or parental education levels were analyzed as independent variables instead of group. This suggested that the observed associations between group and secure/ambivalent/disorganized attachment were independent of ODD symptoms or parental education levels (see Supplementary Materials for detailed results).

### 3.3. Secondary Analyses: Within ADHD Group Analyses

None of the bootstrapped linear regression analyses *within* the ADHD group yielded any significant result. This indicated that age, parental education levels, parent–child relationship quality (as measured by parents’ expressed emotion), and parents’ perceived sense of competence were not associated with any of the attachment representations. For detailed results, see Table 3. The pattern of results did not change when only children with combined or inattentive presentations were considered (see Supplementary Materials Tables S3 and S4 for detailed results for these separate subgroups).

## 4. Discussion

The aim of this study was to investigate whether children with ADHD had more insecure and/or disorganized attachment representations relative to their peers without ADHD. Using story-stem procedures, we found that—in line with our hypothesis—children with ADHD had less secure attachment representations relative to typically developing children. Additionally, children with ADHD had more ambivalent and disorganized attachment representations. No between-group differences were found on avoidant attachment representations. Crucially, we found no effects of ODD symptoms and parental education on any attachment representation, which indicated that the link between insecure and disorganized attachment and ADHD was not explained by these factors.

The link between insecure and disorganized attachment and ADHD could be explained by several mechanisms. First, it may be more challenging to form a secure attachment relationship with children with ADHD relative to typically developing children, because of the inattentive, hyperactive, and impulsive behavior of these children. This may increase parenting stress, interfere with sensitive and responsive parenting behavior, and challenge the parent–child interaction, together challenging parents’ ability to provide a secure base for their child [20,23,49].

Second, early life stress in general, and more specifically the insensitive/unresponsive caretaking behavior that leads to insecure attachment representations, may contribute to the development of ADHD. High levels of early life stress may have direct impact on the maturation of the brain [50,51], potentially contributing to neurodevelopmental psychopathology such as ADHD, whereas sensitive and responsive parenting protects against stress and stimulates children’s brain development [52]. Moreover, prospective studies demonstrate that family risk factors (i.e., parental rejection, unresolved maternal mourning, parent–child relational problems, and mother–child attachment problems) increase the likelihood of developing ADHD later in childhood ([53,54,55], reviewed by [14]). Other compelling evidence for the temporal association between insecure attachment representations and later ADHD has been provided by the English and Romanian Adoptees Study, which shows that children growing up under severe institutional deprivation are more likely to display ADHD symptoms, with linear associations between the duration of deprivation and the severity of ADHD symptoms [56,57].

An explanation for the association between insecure attachment and ADHD could be that children with insecure attachment relationships with their parents constantly devote part of their attention to their safety and their caregiver (e.g., [58]), which impairs the development of their overall self-regulatory capacities (see [13] for an extensive review). A parent who provides secure base support during distress, however, consistently assists the child in regulating emotions and behavior, thereby facilitating the development of self-regulatory functioning [13,59]. Both causal directions (i.e., ADHD challenging secure attachment and insecure attachment contributing to ADHD symptoms) are plausible and it is most likely that challenging child behaviors, parenting problems, and early life stress interact in a synergistic manner during development [14], thereby contributing to the co-occurrence of ADHD and insecure attachment in children.

To better understand which characteristics of children with ADHD were particularly associated with insecure or disorganized attachment representations, we investigated four potentially relevant mechanisms within the ADHD group. Based on earlier work, we reasoned that parent–child relationship quality [60], parents’ perceived sense of competence [61], parental education levels [62], and children’s ODD symptoms [20] were all likely candidates. Our results, however, surprisingly demonstrated that none of these candidate mechanisms were particularly associated with any of the attachment representations within the group of children with ADHD. A potential explanation for the lack of effects on these proposed mechanisms could be that there is large heterogeneity in the causes of attachment problems, and therefore interactions between multiple factors are more likely than the univocal relationships that were currently tested. Another explanation could be that other mechanisms are more relevant in explaining attachment problems in children with ADHD. Here, we propose two related mechanisms that are derived from theoretical models on attachment, and should be empirically tested in future studies.

First, a recent model describing the development of attachment from a learning-theory perspective proposes that secure attachment is learned by reductions in cortisol after caregivers’ support, which is a reinforcer in the safety conditioning of the child, thereby leading to increased trust in the caregiver [63]. More specifically, the contingency of the caregiver’s support (i.e., the likelihood that the caregiver provides support when in stress) is related to the degree of support seeking, showing that expectancy-learning processes play a role in the development of secure attachment [64]. In children with ADHD, these learning processes may be disturbed. More specifically, children with ADHD may be less capable to learn the contingency in the safety signals of their caregivers, as many children with ADHD have an aberrant reward and punishment sensitivity [65,66,67,68]. This implies that children with ADHD typically need larger and more frequent reinforcement to increase their performance/functioning. Regarding the development of attachment, this lower capacity to learn from caregivers’ safety signals would mean children with ADHD may develop less trust in their caregivers. In addition, parents of children with ADHD disproportionately often have elevated AHD symptoms themselves [69], which makes it more challenging to provide consistent and predictable parenting [70].

Second, a related mechanism to explain attachment problems in children with ADHD could come from the differential susceptibility theory, which posits that some children are more susceptible and others are more resilient concerning environmental influences, regardless whether these influences are negative or positive [71,72,73]. Empirical evidence for this theory was provided by a study that showed that VIPP-SD (Video-feedback Intervention to Promote Positive Parenting and Sensitive Discipline) was particularly effective in children with the *DRD4* 7-repeat allele, a polymorphism that was also linked to reward sensitivity and ADHD [74]. This tentatively suggests that a subgroup of children with ADHD is more susceptible to environmental influences, which could explain the increased levels of insecure and disorganized attachment, but would also imply an increased susceptibility to behavioral interventions [75].

### 4.1. Strengths and Limitations

The confidence in our findings is increased by several strengths of the study. Contrary to most studies on ADHD and attachment, we adopted a case-control design with a homogeneous ADHD group in which all children were medication-naive, and used a state-of-the-art story-stem task to measure attachment representations [24]. Moreover, we ascertained that the observed link between ADHD and insecure and disorganized attachment was independent of ODD symptoms and parental education levels.

Although all participating families of children with ADHD actively pursued behavioral treatment, a limitation of our study was that we potentially included a sample of relatively high-functioning families of children with ADHD, as established by average mean intelligence levels in the ADHD group, the low number of high scores on expressed emotions, the fact that none of the children were taking medication, and the fact that we excluded children with comorbid CD. However, it is most likely that between-group differences in attachment representations would rather be larger than smaller if our ADHD group would have been more severely impaired.

### 4.2. Clinical Implications and Future Directions

Based on our findings, we suggest considering both ADHD and attachment when assessing children referred with either ADHD behaviors and/or attachment-related problems. The co-occurrence of ADHD and insecure/disorganized attachment representations is likely explained by several mutually interactive mechanisms (i.e., ADHD behaviors challenging parenting, and early life stress predisposing for ADHD behaviors [12,13,14,23]) and a narrow vision focusing on only one of these aspects is unlikely to capture the full dynamics of the problems these children are encountering.

However, it is yet unclear whether the identification of attachment problems in children with ADHD would lead to different indications for interventions. A hypothesis that awaits empirical scrutiny is that children with ADHD and attachment problems particularly benefit from parenting interventions in which there is an explicit focus on relationship enhancement in addition to behavioral techniques, such as Parent Child Interaction Therapy (PCIT [76]) and VIPP-SD [77]. Crucially, to enhance the learning of children with ADHD, parents’ reactions on support seeking behaviors should be explicit, clear, and frequent [63]. However, a recent meta-analysis on effective elements of behavioral parent training in children with ADHD (regardless of their attachment representations) suggested that foundational behavioral techniques such as stimulus-control and contingency management techniques were associated with the highest effectiveness [78]. In sum, a head-to-head comparison of regular behavioral parent training versus parenting interventions with a focus on relationship enhancement, investigated in children with ADHD with and without attachment problems, seems highly needed. Another recommendation for future intervention studies is to longitudinally investigate whether early treatment of ADHD symptoms fosters the development of secure attachment and, vice versa, whether early interventions aimed at the development of secure attachment representations also prevent later ADHD.

## 5. Conclusions

We demonstrated a link between ADHD and insecure/disorganized attachment representations in school-age children. This study adds to a small but important literature, and there is an urgent need for more studies on the clinical meaning and implications of the association between ADHD and attachment problems.

## Figures and Tables

**Table 1:** Sample characteristics (means and standard deviations, or distribution).

	ADHD (*N* = 74)	TD (*N* = 30)	
Age	8.60 (1.62)	8.03 (1.82)	*t*(102) = −1.57 *n.s.*
Sex (% boys)	64.86%	63.33%	*χ^2^*(1) = 0.02 *n.s.*
IQ	96.09 (13.26)	101.31 (10.81)	*t*(101) = 1.89 *n.s.*
Parental education level	5.26 (1.03)	6.05 (0.58)	*U* = 479.0 ***
Inattention symptoms	7.05 (1.52)	1.53 (1.43)	*U* = 2119.5 ***
Hyp.–Imp. symptoms	6.24 (2.16)	1.27 (1.29)	*U* = 2123.0 ***
ODD symptoms	2.89 (2.14)	0.73 (1.29)	*U* = 1798.5 ***

Note: *** *p* < 0.001, ** *p* < 0.01. For all variables except sex, means are reported, with standard deviations in parentheses. Abbreviations: ADHD = Attention-Deficit/Hyperactivity Disorder, Hyp.–Imp. = Hyperactivity–Impulsivity, *n.s.* = not significant, ODD = Oppositional Defiant Disorder, TD = Typically Developing. Age was analyzed in months but reported in years to enhance readability.

**Table 2:** Attachment representations of children with and without ADHD.

	ADHD (*N* = 74)	TD (*N* = 30)	
Secure	2.73 (1.19)	3.39 (0.75)	*t*(80.37) = 3.49, *p* = 0.001 *
Avoidant	1.90 (0.87)	1.91 (0.56)	*t*(81.90) = 0.26, *p* = 0.793
Ambivalent	1.46 (0.62)	1.23 (0.33)	*t*(92.46) = −2.57, *p* = 0.012 *
Disorganized	1.81 (1.10)	1.32 (0.67)	*t*(79.58) = −3.03, *p* = 0.003 *

Note: Means are reported, with standard deviations in parentheses. Between-group differences were tested in a model with age as covariate. Abbreviations: ADHD = Attention-Deficit/Hyperactivity Disorder; TD = Typically Developing. * Significant after Benjamini–Hochberg correction.

**Table 3:** Bootstrapped linear regression analyses within ADHD group.

	B (95% CI)	*SE*	*β*	*p*
**Secure attachment**				
Age	0.01 (−0.01; 0.03)	0.01	0.09	0.52
Parental education levels	−0.03 (−0.40; 0.37)	0.20	−0.03	0.86
ODD symptoms	−0.01 (−0.15; 0.15)	0.07	−0.02	0.90
Expressed Emotion	0.13 (−0.10; 0.37)	0.11	0.15	0.29
Parenting Sense of Competence	0.01 (−0.02; 0.04)	0.02	0.10	0.43
**Avoidant attachment**				
Age	−0.00 (−0.01; 0.01)	0.01	−0.03	0.85
Parental education levels	0.10 (−0.08; 0.31)	0.10	0.12	0.38
ODD symptoms	−0.02 (−0.15; 0.09)	0.06	−0.06	0.67
Expressed Emotion	0.05 (−0.10; 0.19)	0.08	0.08	0.55
Parenting Sense of Competence	−0.01 (−0.03; 0.02)	0.01	−0.07	0.64
**Ambivalent attachment**				
Age	−0.01 (−0.01; 0.00)	0.00	−0.15	0.15
Parental education levels	0.07 (−0.08; 0.20)	0.07	0.12	0.44
ODD Symptoms	−0.03 (−0.09; 0.05)	0.04	−0.09	0.47
Expressed Emotion	−0.02 (−0.14; 0.11)	0.06	−0.04	0.76
Parenting Sense of Competence	0.01 (−0.01; 0.02)	0.01	0.09	0.55
**Disorganized attachment**				
Age	−0.01 (−0.02; 0.01)	0.01	−0.14	0.41
Parental education levels	−0.04 (−0.36; 0.20)	0.15	−0.04	0.78
ODD Symptoms	0.04 (−0.06; 0.16)	0.06	0.08	0.49
Expressed Emotion	−0.16 (−0.37; 0.07)	0.11	−0.22	0.18
Parenting Sense of Competence	−0.01 (−0.04; 0.02)	0.02	−0.09	0.55

Note: Independent variables were added hierarchically in three blocks, this table depicts the results from the final block containing all predictors. The *β* column represents standardized coefficients; all other statistics are from bootstrapped analyses.

## Data Availability

The data are not publicly available because participating families did not consent for this.

## Supplementary Materials

The following are available online at https://www.mdpi.com/article/10.3390/brainsci11111516/s1, Table S1. Bootstrapped linear regression analyses with ODD symptoms and age as independent variables. Table S2. Bootstrapped linear regression analyses with parental education level and age as independent variables. Table S3. Bootstrapped linear regression analyses within ADHD group (only combined presentation. Table S4. Bootstrapped linear regression analyses within ADHD group (only inattentive presentation.
