# Supplementary material for: Attachment Representations in Children with and without Attention-Deficit/Hyperactivity Disorder (ADHD)
Source: Brain Sci. 2021 Nov 16;11(11):1516. doi: 10.3390/brainsci11111516 (PMC8615467; doi:10.3390/brainsci11111516)
Supplement: Supplementary file 1 [file brainsci-11-01516-s001.zip › brainsci-1447024-supplementary.pdf]

Supplementary Materials

Table S1. Bootstrapped linear regression analyses with ODD symptoms and age as independent variables.

	B (95% ci)	*SE*	*β*	*p*
Secure attachment (R^2=.02)				
ODD symptoms	-.05 (-.15;.05)	.05	-.10	.33
Age	.01 (-.01;.02)	.01	.10	.31
Avoidant attachment (R^2=.01)				
ODD symptoms	-.02 (-.10;.05)	.04	-.06	.57
Age	.00 (-.00;.01)	.00	.07	.40
Ambivalent attachment (R^2=.01)				
ODD symptoms	.00 (-.04;.05)	.02	.01	.93
Age	-.00 (-.01;.00)	.00	-.08	.30
Disorganized attachment (R^2=.05)				
ODD symptoms	.05 (-.05;.15)	.05	.11	.30
Age	-.01 (-.02;.00)	.01	-.18	.11

Table S2. Bootstrapped linear regression analyses with parental education level and age as independent variables.

	B (95% ci)	*SE*	*β*	*p*
Secure attachment (R^2=.03)				
Parental education levels	.16 (-.08;.50)	.15	.14	.27
Age	.00 (-.01;.02)	.01	.07	.48
Avoidant attachment (R^2=.01)				
Parental education levels	.06 (-.09;.19)	.07	.07	.42
Age	.00 (-.00;.01)	.00	.05	.59
Ambivalent attachment (R^2=.00)				
Parental education levels	-.01 (-.10;.07)	.04	-.02	.84
Age	-.00 (-.01;.00)	.00	-.04	.58
Disorganized attachment (R^2=.05)				
Parental education levels	-.15 (-.40;.01)	.11	-.15	.14
Age	-.01 (-.02;.00)	.01	-.14	.21

Table S3. Bootstrapped linear regression analyses within ADHD group (only combined presentation.

	B (95% ci)	*SE*	*β*	*p*
Secure attachment				
Age	.01 (-.01;.03)	.01	.12	.52
Parental education levels	.02 (-.51;.60)	.29	.02	.93
ODD symptoms	-.06 (-.27;.15)	.10	-.11	.58
Expressed Emotion	.03 (-.25;.35)	.15	.04	.83
Parenting Sense of Competence	.00 (-.06;.05)	.03	.02	.93
Avoidant attachment				
Age	-.01 (-.02;.01)	.01	-.16	.34
Parental education levels	.09 (-.17;.36)	.14	.14	.50
ODD symptoms	-.00 (-.14;.12)	.07	-.01	.98
Expressed Emotion	.05 (-.16;.21)	.09	.11	.57
Parenting Sense of Competence	-.01 (-.05;.03)	.02	-.10	.67
Ambivalent attachment				
Age	-.00 (-.01;.01)	.01	-.07	.58
Parental education levels	.05 (-.15;.29)	.11	.09	.61
ODD Symptoms	-.01 (-.11;.10)	.05	-.04	.81
Expressed Emotion	-.00 (-.15;.17)	.08	-.01	.98
Parenting Sense of Competence	.01 (-.02;.04)	.02	.14	.49
Disorganized attachment				

Age	-.01 (-.04;.02)	.01	-.11	.62
Parental education levels	-.06 (-.59;.40)	.25	-.06	.79
ODD Symptoms	.07 (-.11;.27)	.10	.13	.46
Expressed Emotion	-.09 (-.37;.29)	.17	-.12	.60
Parenting Sense of Competence	.01 (-.04;.07)	.03	.07	.74

Note: Independent variables were added hierarchically in three blocks, this Table depicts the results from the final block containing all predictors. The β column represents standardized coefficients, all other statistics are from bootstrapped analyses.

Table S4. Bootstrapped linear regression analyses within ADHD group (only inattentive presentation.

	B (95% ci)	***SE***	***β***	***p***
Secure attachment				
Age	.01 (-.05;.07)	.03	.07	.81
Parental education levels	.29 (-.49;1.15)	.42	.25	.38
ODD symptoms	.11 (-.36;.52)	.22	.20	.54
Expressed Emotion	.41 (-.43;1.35)	.47	.37	.37
Parenting Sense of Competence	.01 (-.09;.08)	.04	.05	.87
Avoidant attachment				
Age	.01 (-.04;.06)	.03	.17	.55
Parental education levels	.07 (-.79;.90)	.41	.07	.76
ODD symptoms	-.23 (-.68;.13)	.19	-.44	.20
Expressed Emotion	.21 (-.69;1.25)	.46	.20	.59
Parenting Sense of Competence	-.01 (-.09;.07)	.04	-.11	.71
Ambivalent attachment				
Age	-.01 (-.04;.01)	.01	-.34	.25
Parental education levels	-.02 (-.52;.34)	.22	-.04	.88
ODD Symptoms	.03 (-.17;.19)	.10	.12	.62
Expressed Emotion	-.22 (-.68;.35)	.26	-.44	.31
Parenting Sense of Competence	-.00 (-.05;.03)	.02	-.06	.88
Disorganized attachment				
Age	-.01 (-.05;.02)	.02	-.36	.37
Parental education levels	-.21 (-.54;.23)	.25	-.37	.24
ODD Symptoms	.05 (-.15;.24)	.11	.17	.64
Expressed Emotion	-.31 (-.69;.16)	.27	-.56	.18
Parenting Sense of Competence	-.01 (-.04;.03)	.02	-.13	.67

Note: Independent variables were added hierarchically in three blocks, this Table depicts the results from the final block containing all predictors. The β column represents standardized coefficients, all other statistics are from bootstrapped analyses.
